# A Murine Commensal Protozoan Influences Host Glucose Homeostasis by Facilitating Free Choline Generation

**DOI:** 10.1128/aem.02413-21

**Published:** 2022-03-22

**Authors:** Yanbo Kou, Liyuan Meng, Shenghan Zhang, Xingping Zheng, Mengnan Liu, Shihong Xu, Qiyue Jing, Hanying Wang, Jinzhi Han, Zhuanzhuan Liu, Yanxia Wei, Yugang Wang

**Affiliations:** a Laboratory of Infection and Immunity, Jiangsu Key Laboratory of Immunity and Metabolism, Department of Pathogenic Biology and Immunology, Xuzhou Medical University, Xuzhou, Jiangsu Province, China; Norwegian University of Life Sciences

**Keywords:** gut microbiota, host metabolism, protozoa, *Tritrichomonas musculis*, gluconeogenesis, choline, choline-utilizing bacteria

## Abstract

Recent progress indicates that the gut microbiota plays important role in regulating the host’s glucose homeostasis. However, the mechanisms remain unclear. Here, we reported that one integral member of the murine gut microbiota, the protozoan *Tritrichomonas musculis* could drive the host’s glucose metabolic imbalance. Using metabolomics analysis and *in vivo* assays, we found that mechanistically this protozoan influences the host glucose metabolism by facilitating the production of a significant amount of free choline. Free choline could be converted sequentially by choline-utilizing bacteria and then the host to a final product trimethylamine N-oxide, which promoted hepatic gluconeogenesis. Together, our data reveal a previously underappreciated gut eukaryotic microorganism by working together with other members of microbiota to influence the host’s metabolism. Our study underscores the importance and prevalence of metabolic interactions between the gut microbiota and the host in modulating the host’s metabolic health.

**IMPORTANCE** Blood glucose levels are important for human health and can be influenced by gut microbes. However, its mechanism of action was previously unknown. In this study, researchers identify a unique member of the gut microbes in mice that can influence glucose metabolism by promoting the host’s ability to synthesis glucose by using nonglucose materials. This is because of its ability to generate the essential nutrient choline, and choline, aided by other gut bacteria and the host, is converted to trimethylamine N-oxide, which promotes glucose production. These studies show how gut microbes promote metabolic dysfunction and suggest novel approaches for treating patients with blood glucose abnormality.

## INTRODUCTION

The gut microbiota has been recognized as an important regulator of the host’s glucose homeostasis, potentially impacting the development of metabolic disorders such as obesity and its related metabolic syndrome (1). However, the underlying mechanism remains incompletely understood. The gut microbiota consists of a diverse community of microorganisms, including viruses, prokaryotic bacteria, eukaryotic fungi, helminths, and protists (2). It is increasingly evident that a constitutive protistic microbiota acts as an integral part of the mammalian gut microbiota. The impact of these eukaryotic protists on the host metabolism is currently unclear. Recently, several reports suggest that some rodent protozoa belong to the parabasalid *Tritrichomonas* spp. can modulate the intestinal immune landscape and barrier functions at least in part through the production of extracellular succinate (3–6). In particular, the protozoan *T. musculis* can activate the host epithelial inflammasome, confer protection from intestinal bacterial infections, exacerbate T cell-driven colitis, and promote the progression of colorectal tumors (7). Several laboratories, including our own, have further shown that intestinal protozoa, including *T. musculis*, can modulate the mammalian gut microbiota landscape (8–11). Whether commensal protozoa have any influence on host metabolism is currently underappreciated.

In this study, we describe the critical contribution of *T. musculis* in shaping host glucose homeostasis. We show that intestinal colonization with *T. musculis* leads to a boost of free choline production, which in turn can be further metabolized by choline-utilizing bacteria, followed by the host, to produce an end product that can enhance gluconeogenesis. These results uncover a potential pathway of the gut microbial community in modulating the host glucose metabolism.

## RESULTS

### A murine symbiotic protozoan promotes gluconeogenesis.

**FIG 1:**
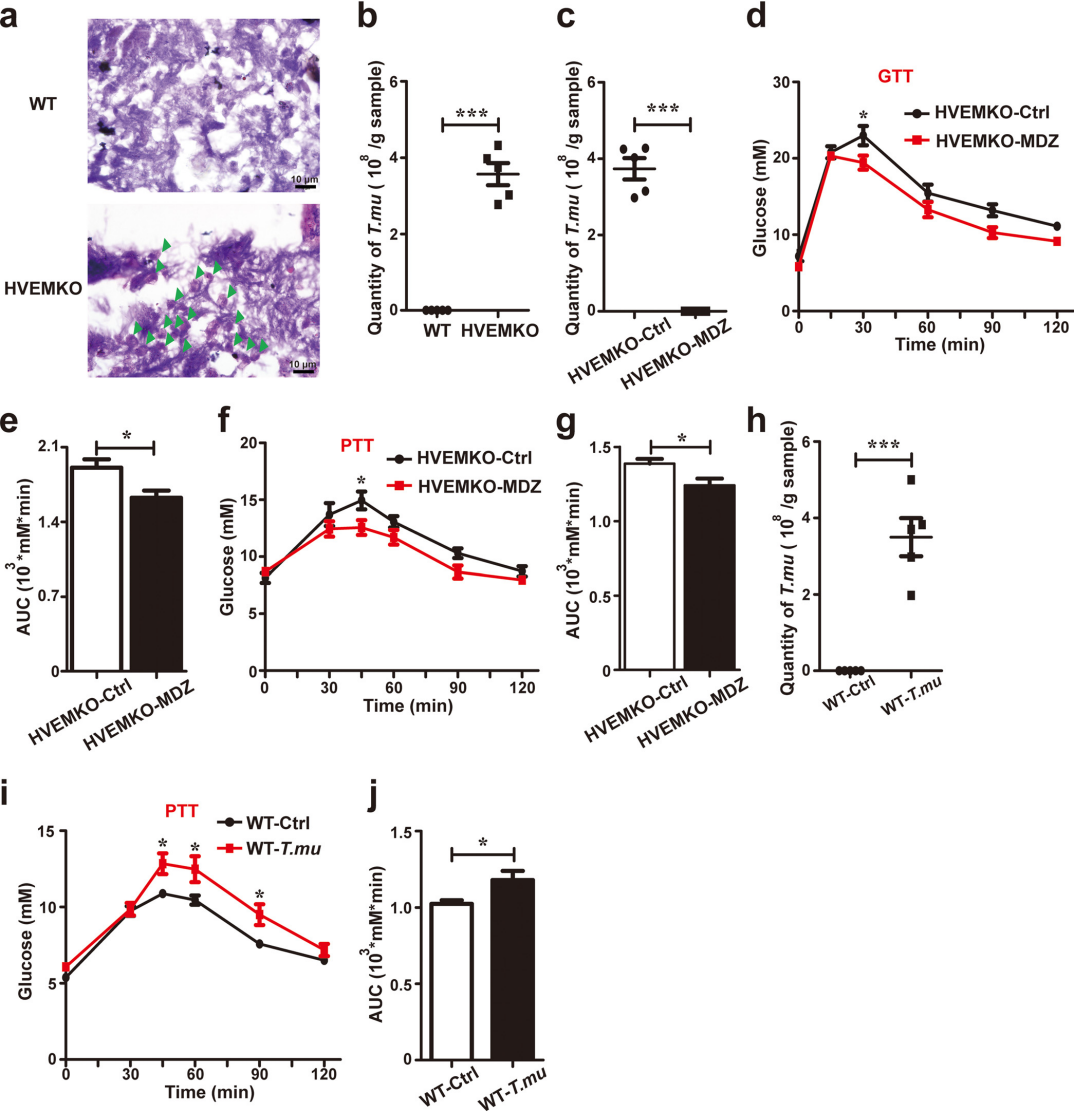
The murine protozoan *T. musculis* promotes gluconeogenesis. (a) The cecal contents of WT and HVEM KO mice were fixed, stained with hematoxylin and eosin, and visualized microscopically. Green arrowheads indicate protozoa. Scale bar, 10 μm; *n* = 3. (b) Total number of *T. musculis* protozoa in the cecal contents of the indicated mice. *n* = 5. (c to g) Eight-week-old male HVEM KO mice were treated with vehicle or 2.5 g/liter metronidazole (MDZ) in drinking water for 1 week. The number of *T. musculis* presented in the cecal content was enumerated before (ctrl) and after MDZ treatment (c). Glucose tolerance testing (GTT) (d and e) or pyruvate tolerance testing (PTT) (f and g) were performed. AUC, the area under the curve. *n* = 5 mice per group. (h to j) *T. musculis* protozoa isolated from HVEM KO mice were transferred to *T. musculis*-free WT mice (1 × 10^6^/mouse). The number of *T. musculis* colonized in the WT recipient mice was calculated (h), and PTT was performed (i and j). *n* = 5 mice per group. The experiments were repeated at least two times. The data represent means ± the SEM. *, *P < *0.05; **, *P < *0.01. *T.mu*, *T. musculis*.

We initially observed that our in-house bred specific-pathogen-free herpesvirus entry mediator (HVEM)-deficient mice (*HVEM^−/−^*) had higher fasting blood glucose, worse glucose intolerance during glucose tolerance testing (GTT), and increased gluconeogenesis during pyruvate tolerance testing (PTT) compared to wild-type (WT) mice obtained from Vital River Laboratory Animal Technology (VRL) (see Fig. S1 in the supplemental material). Subsequent microscopic analysis of fecal material revealed the presence of numerous single-celled protozoa in *HVEM^−/−^* but not in VRL WT mice (Fig. 1a and b). Molecular PCR, followed by DNA sequencing, at the 18S and ITS rDNA locus identified the protozoa were murine commensal *T. musculis*, which we have characterized previously (8).

To determine whether the disturbed glucose homeostasis observed in *HVEM^−/−^* mice was related to *T. musculis* colonization or not, we eradicated *T. musculis* from the mice by adding metronidazole (2.5 g/L) to their drinking water for 1 week. This treatment eliminated *T. musculis* from the gut (Fig. 1c), and concomitantly improved glucose intolerance, and reduced gluconeogenesis in *HVEM^−/−^* mice (Fig. 1d to g). However, the same metronidazole treatment was not able to temper gluconeogenesis in *T. musculis*-free WT mice from VRL (see Fig. S2a and b). Furthermore, depleting bacterial microbiota but sparing *T. musculis* protozoa in *HVEM^−/−^* mice by using a cocktail of broad-spectrum antibiotics (vancomycin, ampicillin, and neomycin) lacking metronidazole failed to affect glucose homeostasis (Fig. S2c to e).

To further corroborate *T. musculis* colonization influencing glucose homeostasis, we isolated *T. musculis* from *HVEM^−/−^* mice and transferred them to *T. musculis*-free WT mice from VRL. *T. musculis* colonization significantly changed the gut microbiota landscape (see Fig. S3), and concomitantly elevated gluconeogenesis in the recipient mice (Fig. 1h to j). In aggregate, these findings suggest that the murine commensal protozoan *T. musculis* can promote gluconeogenesis.

### Succinate does not enhance gluconeogenesis.

We next investigated how *T. musculis* might affect the host’s blood glucose homeostasis. We hypothesized that *T. musculis*-derived metabolites might be the key modulators. *Tritrichomonas* is known generally to be able to produce succinate by fermentative pyruvate oxidation in its hydrogenosomes (12). We confirmed via mass spectrometric analysis that *T. musculis* cultured *in vitro* for 2 days was indeed able to produce succinate (see Fig. S4a). Furthermore, *T. musculis*-free WT mice once colonized with *T. musculis* acquired higher levels of cecal and serum succinate (see Fig. S4a). Thus, we explored whether succinate could promote gluconeogenesis. Succinate in drinking water did not significantly promote gluconeogenesis (see Fig. S4b). Further, succinate receptor-deficient (*Sucnr1^−/−^*) mice, which are unable to sense extracellular succinate, had a normal level of gluconeogenesis compared to WT mice (Fig. S4c). These data together do not support *T. musculis*-derived succinate as a potential candidate in promoting host gluconeogenesis.

### *T. musculis* facilitates free choline release.

**FIG 2:**
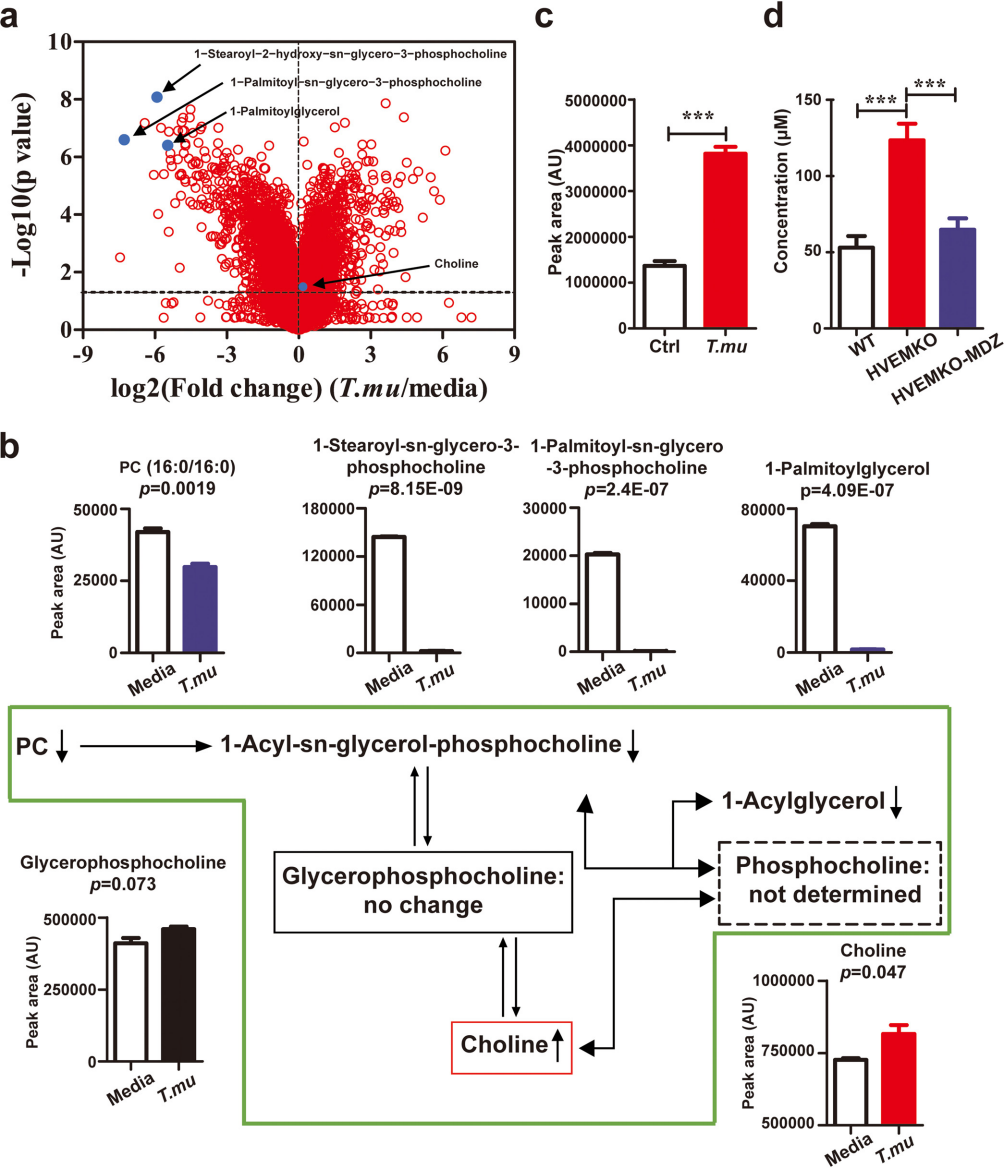
*T. musculis* facilitates free choline release. (a) Volcano plot of the metabolite levels in 2-day-old *T. musculis*-cultured supernatant versus untouched culture media. Each red dot represents a unique metabolite. Blue dots indicate several selected metabolites that are related to choline metabolism. *n* = 3 per group. (b) Choline metabolic pathway and concentrations of selected metabolites in 2-day-old *T. musculis*-cultured supernatant and untouched media. Each up- or downward arrow indicates that the specific metabolite concentration in the medium is increased or decreased after *T. musculis* culture, respectively. (c) Free choline levels in cecal contents of *T. musculis*-colonized and *T. musculis*-free control mice. *n* = 10 per group. (d) Free choline concentrations in the sera of WT, HVEM knockout (KO), and HVEM KO mice that had undergone 1-week metronidazole (MDZ) treatment to remove *T. musculis*. *n* = 4 to 9/group. The data represent means ± the SEM. *, *P < *0.05; **, *P < *0.01; ***, *P < *0.001. *T.mu*, *T. musculis*.

To assess what other metabolites that *T. musculis* might produce, we collected *in vitro* 2-day *T. musculis*-cultured supernatant and did an untargeted metabolomics analysis. The most prominent feature we found was that *T. musculis* culture led to depletions of large portions of choline precursors from the media, including phosphatidylcholines (PCs) (e.g., PC [16:0/16:0]) and lyso-PCs (e.g., 1-stearoyl-2-hydroxy-*sn*-glycero-3-phosphocholine and 1-palmitoyl-*sn*-glycero-3-phosphocholine), and concomitantly there was a net increase of free choline in the supernatant (Fig. 2a and b), suggesting that *T. musculis* might facilitate processing choline precursors to release free choline. Likewise, transferring *T. musculis* to WT mice increased free choline concentrations in the cecal contents (Fig. 2c), and concomitantly the serum levels of choline-derived metabolites (e.g., glycerophospholipids and betaine) were all augmented (see Fig. S5). Furthermore, eradication of *T. musculis* from *HVEM^−/−^* mice by metronidazole reduced the serum choline levels by more than 50% (Fig. 2d). Together, our data imply that *T. musculis* may greatly facilitate free choline production.

### Free choline may need to be further processed by choline-utilizing bacteria to modulate gluconeogenesis.

**FIG 3:**
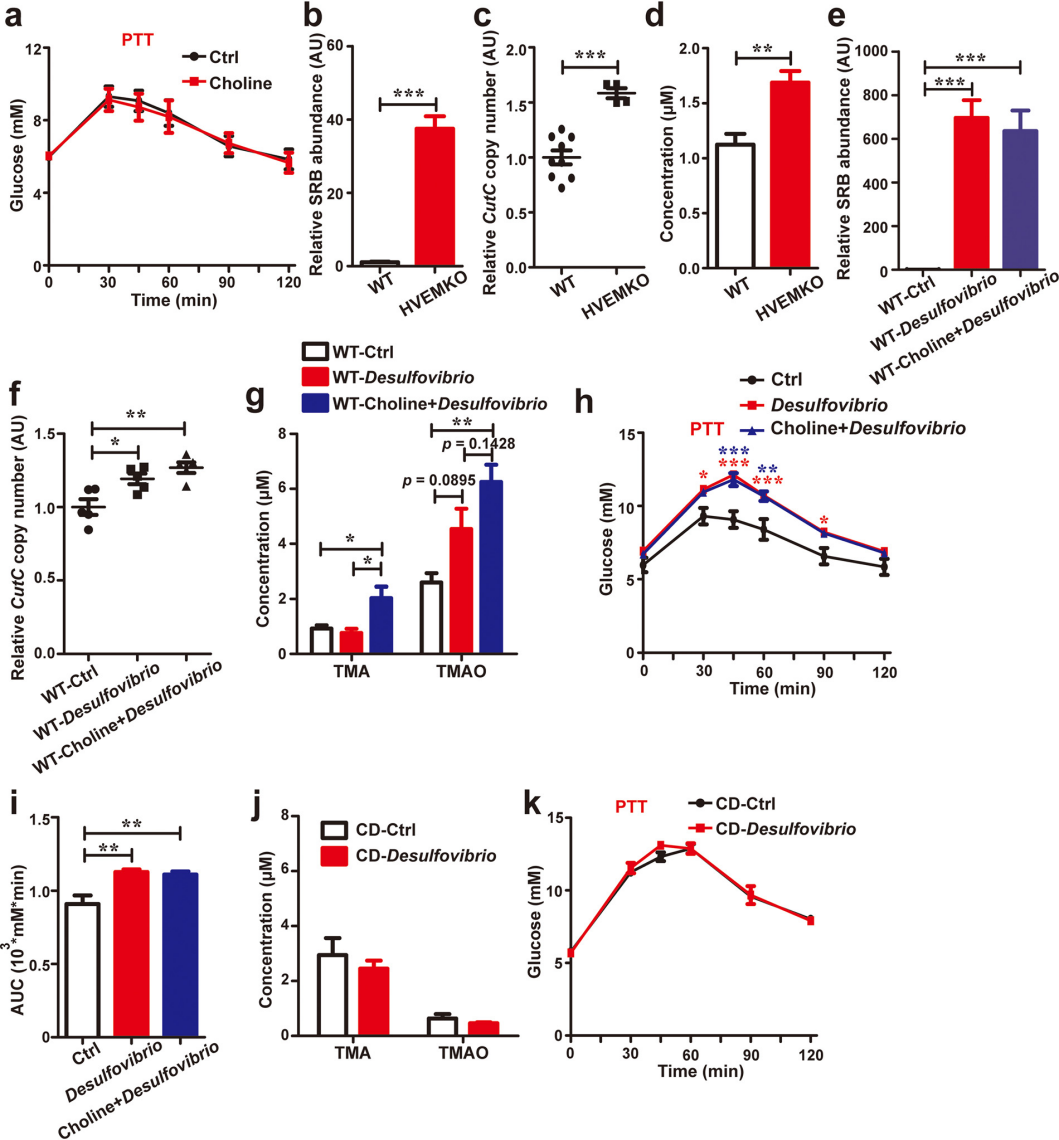
Free choline needs to be processed by choline-utilizing bacteria to promote gluconeogenesis. (a) Eight-week-old male *T. musculis-free* WT mice were treated with vehicle or 1 g/liter free choline in drinking water for 1 week. Pyruvate tolerance testing (PTT) was performed. *n* = 5 to 10 mice/group. (b) Relative abundance of the choline-utilizing bacteria *Desulfovibrio* in the cecal contents of WT and HVEM KO mice. (c) Relative abundances of bacteria expressing the choline trimethylamine (TMA)-lyase (*cutC*) gene in the cecal contents of the indicated mice. (d) Serum levels of TMA in the WT and HVEM KO mice. (e to h) *D. vulgaris* collected from *in vitro* culture were administered via oral gavage into the *T. musculis*-free WT mice. The mice were then treated with vehicle or 1 g/liter free choline in drinking water for 1 week. The relative abundances of *Desulfovibrio* (e) and *cutC*-positive bacteria (f) in the cecal contents of the indicated groups of mice were determined, serum TMA and TMAO levels were monitored (g), and PTT was performed (h and i). *n* = 5 to 10 mice/group. The red and blue asterisks in panel h represent statistical significance of the *D. vulgaris* group (red) or the *D. vulgaris*-plus-choline group (blue) when compared to the control group, respectively. (j and k) Eight-week-old male *T. musculis*-free WT mice that were originally fed on a normal chow diet were orally administered *D. vulgaris* and then placed on a choline-deficient (CD) diet for 1 week, the serum TMA and TMAO levels were determined (j), and PTT was performed (k). *n* = 5 mice per group. The experiments were repeated at least twice. The data represent means ± the SEM. *, *P < *0.05; **, *P < *0.01; ***, *P < *0.001.

To test whether free choline can induce gluconeogenesis, we provided extra free choline (1 g/L) in drinking water to WT mice. We initially did not find any impact on gluconeogenesis by simply providing more free choline to the mice (Fig. 3a). We then considered the possibility of a requirement of choline transformation by the gut microbiota, as it is known that free choline can be converted to trimethylamine (TMA) by some anaerobic bacteria encoding the choline utilization (*cut*) gene cluster, such as *Desulfovibrio* (13). Indeed, there was a relatively low abundance of choline-utilizing bacteria, including *Desulfovibrio*, in the WT mice compared to *HVEM^−/−^* mice (Fig. 3b and c). The serum level of TMA in the WT mice was also lower than that in the *HVEM^−/−^* mice (Fig. 3d). Furthermore, choline supplementation in WT mice failed to increase serum TMA level (see Fig. S6), suggesting relatively low activity of bacterial choline conversion in these animals. To increase the number of choline-utilizing bacteria, we administered *in vitro* cultured *Desulfovibrio vulgaris*, which encodes choline TMA-lyase (*cutC*) gene (see Fig. S7), to the WT mice via oral gavage. This manipulation increased the abundance of *Desulfovibrio* in the WT recipient host significantly (Fig. 3e), and in correspondence, the relative abundance of total *cutC*-positive bacteria was increased as well (Fig. 3f). Compared to the control mice, *D. vulgaris-*transferred mice, when fed with extra free choline in drinking water, had increased serum levels of both TMA and its derivative trimethylamine-*n*-oxide (TMAO) (Fig. 3g), and in the meantime, the gluconeogenesis was also elevated (Fig. 3h and i). Interestingly, *D. vulgaris* transfer alone even without adding extra choline in drinking water was already effective in promoting gluconeogenesis (Fig. 3h and i). We speculated that, even without extra choline in drinking water, *Desulfovibrio* might still be able to access dietary choline to facilitate its conversion, which might be sufficient to promote gluconeogenesis. Consistent with this, *D. vulgaris* transfer alone had a trend to increase serum TMAO levels when fed with a normal chow diet (Fig. 3g), although this did not yet reach statistical significance. However, when we switched the animal diet to a choline-deficient diet, this trend no longer existed (Fig. 3j), and it also failed to promote gluconeogenesis (Fig. 3k). These observations overall suggest that to influence gluconeogenesis free choline derived from both the diet and the choline-releasing organisms (e.g., *T. musculis*) may need to be further processed by choline-utilizing commensal bacteria.

### The enhancement of gluconeogenesis by *T. musculis* depends on choline downstream metabolism.

**FIG 4:**
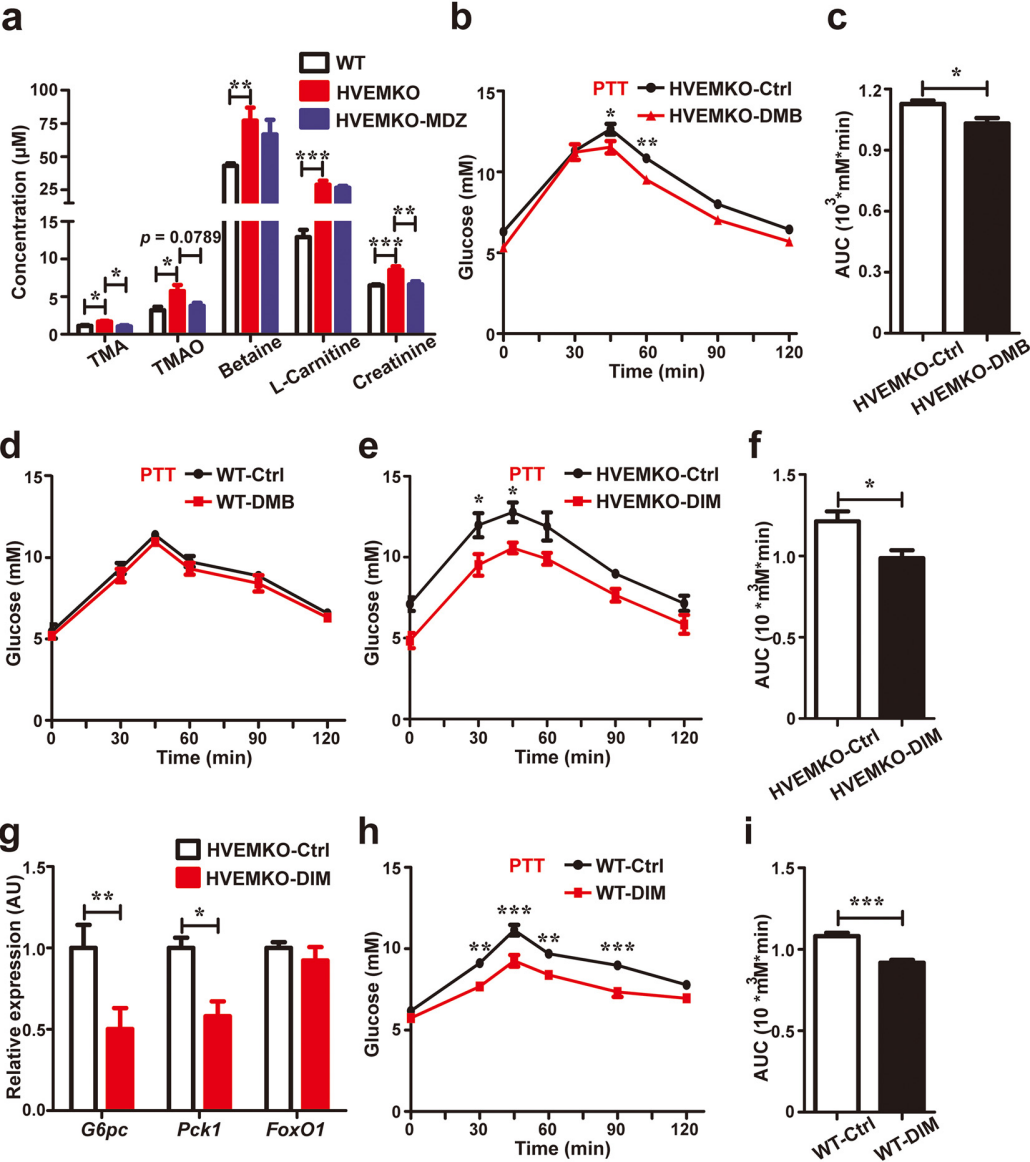
Enhancement of gluconeogenesis by *T. musculis* depends on choline downstream metabolism. (a) Serum levels of several selected choline derivatives in WT, HVEM KO, and HVEM KO mice that had been treated with metronidazole for 1 week. *n* = 4 to 9 mice/group. (b to d) Eight-week-old male *T. musculis*-bearing HVEM KO mice (b and c) or *T. musculis*-free WT mice (d) were treated with 1% 3,3-dimethyl-1-butanol (DMB) in drinking water for 1 week, and PTT was then performed. AUC, the area under the curve. *n* = 5 mice/group. (e and f) Eight-week-old male HVEM KO mice were placed on a standard chow diet with or without supplementation of 0.25% 3,3′-diindolylmethane (DIM) added to the diet for 4 weeks, and then PTT was performed. AUC, the area under the curve. *n* = 5 mice/group. (g) Relative hepatic expressions of the indicated key genes involved in gluconeogenesis in HVEM KO mice with or without DIM intervention. *n* = 5 mice/group. (h and i) Eight-week-old male *T. musculis*-free WT mice were placed on a standard chow diet with or without supplementation of 0.25% DIM added to the diet for 4 weeks, and then PTT was performed. AUC, the area under the curve. *n* = 5 mice/group. The experiments were repeated at least two times. The data represent means ± the SEM. *, *P < *0.05; **, *P < *0.01; ***, *P < *0.001.

Free choline inside the intestinal cavity can be converted to TMA by some anaerobic bacteria (13). TMA can further be absorbed into the host’s bloodstream and converted to TMAO via liver flavin-containing monooxygenase 3 (FMO3) (14). TMAO was reported to be able to facilitate hepatic gluconeogenesis (15). Therefore, we assessed whether the choline-TMA-TMAO metabolic pathway was active in *T. musculis*-colonized *HVEM^−/−^* mice. The serum levels of choline and its downstream metabolites, including TMA and TMAO, were all increased in *HVEM^−/−^* mice compared to *T. musculis*-free WT mice (Fig. 4a and Fig. 2d). Further, the serum TMA level in *HVEM^−/−^* mice reverted to the normal WT level after metronidazole treatment (Fig. 4a). The serum TMAO level in *HVEM^−/−^* mice also had a trend to be reduced by metronidazole treatment but failed to reach a statistical significance (Fig. 4a). To assess whether the enhanced gluconeogenesis observed in *HVEM^−/−^* mice was dependent on the choline-TMA-TMAO metabolic pathway, we utilized a small molecular inhibitor 3,3-dimethyl-1-butanol (DMB) to inhibit the bacterial conversion of choline to TMA *in vivo* (16). DMB treatment was effective in reducing gluconeogenesis in *HVEM^−/−^* mice (Fig. 4b and c) but has no impact in *T. musculis*-free WT mice (Fig. 4d).

Since the phytochemical 3,3′-diindolylmethane (DIM) can inhibit FMO3 activity (17), we also placed *HVEM^−/−^* mice on a diet with or without DIM supplementation. DIM treatment reduced gluconeogenesis and inhibited hepatic expression of gluconeogenic genes glucose-6-phosphatase (*G6pc*) and phosphoenolpyruvate carboxykinase 1 (*Pck1*) in *HVEM^−/−^* mice (Fig. 4e to g). Interestingly, DIM treatment was also effective in reducing the basal level of gluconeogenesis in *T. musculis*-free WT mice (Fig. 4h to i), suggesting that the basal activity of FMO3, which can convert TMA to TMAO, may participate in modifying normal glucose metabolism.

**FIG 5:**
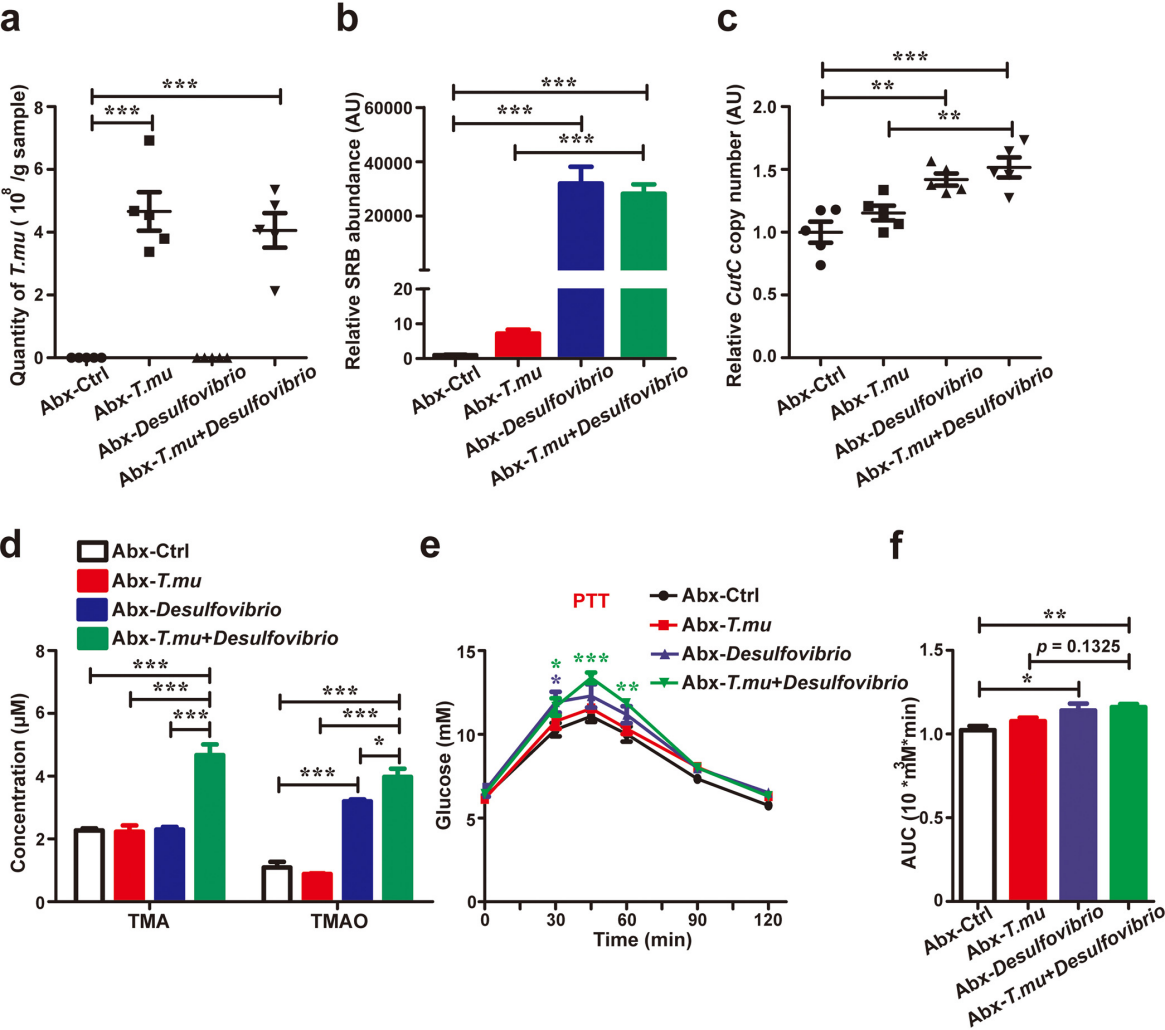
*T. musculis* requires choline-utilizing bacteria to affect the host’s glucose homeostasis. (a to f) Eight-week-old *T. musculis*-free WT male mice were treated with a cocktail of broad-spectrum antibiotics (Abx, ampicillin, neomycin, metronidazole, and vancomycin in drinking water) for 1 week, rested for another week, and then administered vehicle (ctrl), *T. musculis*, *D. vulgaris*, or *T. musculis* plus *D. vulgaris* via oral gavage, respectively. After an additional week, the numbers of *T. musculis* (a), the relative abundance of *Desulfovibrio* (b), and the numbers of *cutC*-positive bacteria (c) in the cecal contents of the indicated mice were determined, the serum levels of TMA and TMAO were monitored (d), and pyruvate tolerance testing (PTT) was performed (e and f). AUC, the area under the curve. *n* = 5 mice/group. The green and blue asterisks in panel e represent the statistical significance compared to the *T. musculis*-plus-*D. vulgaris* group (green) or the *D. vulgaris* group (blue) versus the control group, respectively. The data represent means ± the SEM. *, *P < *0.05; **, *P < *0.01; ***, *P < *0.001. *T.mu*, *T. musculis*.

Our data so far have demonstrated that the choline downstream metabolism is critical to influencing glucose homeostasis, but whether free choline-producer *T. musculis* requires choline-utilizing bacteria to affect gluconeogenesis remains to be determined. To this end, we transferred *T. musculis* with or without *D. vulgaris* to WT mice whose microbiota had been wiped out by antibiotics and confirmed their colonization after transfer (Fig. 5a to c). We found that *T. musculis* transfer alone at this condition was not sufficient to either increase serum TMA and TMAO levels or promote gluconeogenesis (Fig. 5d to f). However, when combined with *D. vulgaris* together, *T. musculis* colonization could then greatly increase serum TMA and TMAO levels and promote more gluconeogenesis (Fig. 5d to f). Again, *D. vulgaris* transfer alone was already effective in promoting gluconeogenesis and showed signs of facilitating choline conversion indicated by an increase of serum levels of TMAO (Fig. 5d to f), probably via processing dietary derived choline. The data collectively indicate that the impact of *T. musculis* on host gluconeogenesis is dependent on the activities of downstream choline metabolism.

## DISCUSSION

It is known that the gut microbiota influences its host’s systemic glucose homeostasis (18), but the details remain unclear. In this investigation, we used metabolite analysis and *in vivo* assays to show how a mouse eukaryotic commensal protozoan *T. musculis* may regulate glucose homeostasis. Our work suggests that *T. musculis* can facilitate free choline liberation, and in turn, free choline can be further processed by the choline-utilizing microorganisms and finally by the host to produce a product that can modulate glucose homeostasis through hepatic gluconeogenesis.

The most abundant form of choline available in both the human diet and the human body is not free choline but instead PC (19, 20). A previous study indicates at least one way to obtain free choline for the host is through bacterial hydrolysis of PC (21). Our work suggests that not only choline-degrading gut bacteria but probably a larger subset of gut microorganisms, including eukaryotic protists, can metabolize PC. The overall contribution of the gut protozoan *T. musculis* to the total free choline pool in the host can be very significant. In our experiment, *T. musculis* deletion by metronidazole resulted in more than 50% reduction of the serum choline level. However, the exact metabolic pathway of PC in *T. musculis* requires future investigation. Patients with ulcerative colitis have a significant reduction of PC in the mucosal barrier (22). Supplementation of PC can restore mucosal integrity in inflammatory bowel disease patients (23). Our results raise the possibility that PC-consuming microorganisms like *T. musculis* may contribute to barrier dysfunction if intestinal homeostasis is broken. Indeed, *T. musculis* exacerbated T cell-driven experimental colitis in mice (7). Manipulating the gut microbiota, including the members of eukaryotic protists, may hold promise to control the progression of inflammatory bowel disease.

Our data suggest that the way choline influences glucose metabolism may rely on downstream choline metabolism by both the microbiota and the host. Free choline inside the host’s internal tissues can be transformed to either the neurotransmitter acetylcholine, the membrane lipids PC and sphingomyelin, or the methyl donor betaine (24), and choline inside the intestinal cavity can also be transformed to TMA, which is further oxidized in the liver to TMAO. Thus, the net influence of free choline on host metabolism is probably dependent on a balance between the microbial and the host’s metabolic activities. The mechanisms by which choline is linked to host metabolic health remain to be fully explored. Choline injection into rats can increase insulin, glucagon, and catecholamine, all of which can influence glucose homeostasis (25, 26).

Blood TMAO level has been suggested as a prognostic marker for cardiovascular and cerebrovascular diseases (27, 28). However, multiple confounding factors (e.g., intestinal microbiota, diet, age, body mass, sex hormones, renal clearance, FMO3 expression, and genetic background) can influence circulating TMAO levels and whether there is a population-wise consistent pathogenic threshold of TMAO remains a matter of debate. Recently, TMAO was also found to be able to promote hepatic gluconeogenesis via protein kinase R-like endoplasmic reticulum kinase (15). Our work is consistent with the previous work and further reveals that phospholipid metabolism by the gut microorganisms plays important role in adjusting the host’s metabolic homeostasis. Our data support the gut microbiota as a promising target for therapeutic intervention for diseases linked to the host’s metabolic disturbance.

## MATERIALS AND METHODS

### Animal studies.

All animal work was conducted with the formal approval of the animal care committee of Xuzhou Medical University and was performed at Xuzhou Medical University’s Laboratory Animal Centre followed all the mandatory laboratory health and safety procedures. Mice were housed in a temperature-controlled room (22 ± 2°C) and subjected to a 12-h light/dark cycle, with free access to water and food. C57BL/6 male mice (6 to 8 weeks old) were purchased from Vital River Laboratory Animal Technology. *HVEM^−/−^* and *SUCNR1^−/−^* mice in this study were both on a C57BL/6 background. The *HVEM^−/−^* mice were kindly provided by Yang-Xin Fu (University of Texas Southwestern) and were bred in-house for more than 10 generations. The *SUCNR1^−/−^* mice were purchased from Cyagen Biosciences. Mice were fed a normal chow diet or a choline-deficient diet (Keao Xieli Feed Co.). The ingredients of the choline-deficient diet were as follows: casein, 233.6 g/kg; l-cysteine, 3.5 g/kg; corn starch, 85 g/kg; maltodextrin, 116.8 g/kg; sucrose, 201.8 g/kg; cellulose, 58.4 g/kg; soybean oil, 29.2 g/kg; lard, 207.3 g/kg; S10026 polymineral, 11.7 g/kg; calcium hydrogen phosphate, 15.2 g/kg; calcium carbonate, 6.4 g/kg; potassium citrate monohydrate, 19.3 g/kg; V10001 multivitamin, 11.7 g/kg; and antioxidant, 0.1 g/kg.

Whenever needed, mice were fed in the drinking water with either 1% (vol/vol) 3,3-dimethyl-1-butanol (DMB; Sigma-Aldrich, catalog no. 183105), 0.1% (wt/vol) choline (Sigma-Aldrich, catalog no. C7527), or 2 to 5% disodium succinate (Macklin Biochemical, catalog no. S818135) or were fed in the diet with 0.25% (wt/wt) 3,3′-diindolylmethane (DIM; Sigma-Aldrich, catalog no. D9568). To eliminate *T. musculis*, 1 g/L metronidazole (Sangon Biotech, catalog no. A600633) was added to the drinking water for a week. To deplete commensal bacteria other than *T. musculis*, an antibiotic cocktail lacking metronidazole (1 g/L neomycin [Sigma-Aldrich, catalog no. N6386], 0.5 g/L vancomycin [Sangon Biotech, catalog no. A600983], or 1 g/L ampicillin [Sangon Biotech, catalog no. A100741]) was added to the drinking water for 1 week. For Fig. 5, an antibiotic cocktail containing 1 g/L neomycin, 0.5 g/L vancomycin, 1 g/L ampicillin, and 1 g/L metronidazole was used. For *T. musculis* transfer, each mouse received 1 × 10^6^ *T. musculis* via oral gavage. For *D. vulgaris* transfer, a dose of 10^8^ CFU per mouse was administered via oral gavage once every other day for a total of 1 week, while the control mice were given an equal volume of phosphate-buffered saline (PBS).

### Isolation and culture of *T. musculis*.

The cecal contents of *HVEM^−/−^* mice were harvested into sterile PBS and filtered three times through a 100-μm cell strainer. The filtrate was centrifuged at 200 × *g* for 5 min at 4°C. The pellet was washed twice with PBS. *T. musculis* enriched in the pellet was further purified using a 40%/80% Percoll gradient. For *T. musculis* culture, the isolated *T. musculis* was suspended with BHI broth (Oxoid, catalog no. CM1135) supplemented with a cocktail of broad-spectrum antibiotics, including 100 mg/mL streptomycin (Sangon Biotech, catalog no. A100382), 100 U/mL penicillin (Sangon Biotech, catalog no. A613460), 50 mg/mL vancomycin (Sangon Biotech, catalog no. A600983), 10 mg/mL ciprofloxacin (Sangon Biotech, catalog no. A600310), 20 mg/mL gentamicin (Sangon Biotech, catalog no. A506614), and 0.5 mg/mL amphotericin B (Sangon Biotech, catalog no. 171375). After suspension, *T. musculis* was then incubated in an anaerobic workstation (Don Whitley Scientific) at 37°C for 2 days.

### *D. vulgaris* culture.

Desulfovibrio vulgaris (CGMCC 1.5190) was purchased from the China Common Microbial Strain Preservation and Management Center. *D vulgaris* was anaerobically cultured at 37°C in *Desulfovibrio* (Postgate) medium containing KH_2_PO_4_ (0.5 g/L), NH_4_Cl (1 g/L), Na_2_SO_4_ (1 g/L), MgSO_4_⋅7H_2_O (2 g/L), CaCl_2_⋅2H_2_O (0.1 g/L), yeast extract (1 g/L), resazurin (1 mg/L), sodium lactate (2 g/L), FeSO_4_⋅7H_2_O (0.5 g/L), sodium thioglycolate (0.1 g/L), and vitamin C (0.1 g/L) (pH 7.8).

### Quantification of *T. musculis* protozoa in the cecal content.

Cecal content was suspended in PBS (0.5 mL/50 mg). The protist presented was then counted microscopically using a hemocytometer as previously reported (7, 8).

### Fasting blood glucose measurements, glucose tolerance test, and pyruvate tolerance test.

Mice were fasted overnight for fasting blood glucose measurement and glucose/pyruvate tolerance tests. For glucose or pyruvate tolerance tests, a 2-g/kg dose of glucose or pyruvate was injected intraperitoneally. Insulin tolerance tests were conducted after 6 h of fasting, and a 0.75-U/kg dose of insulin (Biogems-PeproTech, catalog no. 10-365) was administered intraperitoneally. Blood glucose was measured with a glucometer (Accu-Chek Active [Roche] or ONETOUCH UltraEasy [Johnson & Johnson]) using whole blood from the tails. The area under the curve (AUC) was calculated using the trapezoid rule in Prism (GraphPad Software). The total peak area from baseline was determined for each mouse, and the baseline was defined as the basal blood glucose concentration (time zero).

### Untargeted metabolomics analysis.

This was performed by Shanghai Applied Protein Technology (Shanghai, China). For the cell culture samples, they were cleared by centrifugation at 1,000 × *g* and 4°C for 3 min before mass spectrometry analysis. For the serum samples, every sample was diluted with four times the volume of prechilled methanol-acetonitrile solution (1:1, vol/vol) and mixed well by vortexing. After 10 min of incubation at –20°C, the mixture was centrifuged at 14,000 × *g* and 4°C for 15 min, and the supernatant was collected and vacuum-dried. Finally, the acetonitrile aqueous solution (acetonitrile-water [1:1, vol/vol]) was added for reconstitution before mass spectrometry analysis. For the cecal content samples, every 60 mg of cecal contents was mixed with 200 μL of ultrapure water and homogenized; then, 800 μL of methanol-acetonitrile (1:1, vol/vol) was added. After sonication at low temperature for 30 min, the sample was incubated at –20°C for another 60 min. To precipitate proteins, the sample was centrifuged at 14,000 × *g* and at 4°C for 15 min. The supernatant was then collected and freeze-dried. All samples were separated by ultra high performance liquid chromatography (UHPLC) and analyzed by using a Triple TOF 5600 mass spectrometer (AB SCIEX). The UHPLC separation was carried out using the Agilent 1290 Infinity LC UHPLC System (Agilent Technologies). For Q-TOF mass spectrometry (MS) analysis, electrospray ionization (ESI)-positive ion and -negative ion modes were used for detection.

### Targeted UHPLC-MS/MS of choline and its downstream metabolites measurements.

Targeted UHPLC-MS/MS of choline and its downstream metabolites measurements was performed by Shanghai Biotree Biomedical Technology Co., Ltd. (Shanghai, China). The serum was mixed with a 4× volume of 0.1% formic acid aqueous solution and a 20× volume of acetonitrile (0.1% formic acid, containing isotopically labeled internal standard mixture), vortexed for 30 s, and then sonicated for 15 min in an ice-water bath, followed by incubation at –40°C for 60 min. After centrifugation at 12,000 × *g* and 4°C for 15 min, an 80-μL aliquot of the clear supernatant was transferred to an autosampler vial for UHPLC-MS/MS analysis. UHPLC separation was carried out using an Agilent 1290 Infinity II Series UHPLC System (Agilent Technologies) equipped with ACQUITY UPLC BEH Amide (100 × 2.1 mm, 1.7 μm; Waters). An Agilent 6460 triple-quadrupole mass spectrometer (Agilent Technologies) equipped with an AJS ESI interface was applied for assay development. The multiple reaction monitoring (MRM) parameters for each of the targeted analytes were optimized using flow injection analysis by injecting the standard solutions of the individual analytes into the API source of the mass spectrometer. Agilent MassHunter Work Station Software (B.08.00; Agilent Technologies) was used for MRM data acquisition and processing.

### Hematoxylin and eosin staining.

The cecal samples were fixed in 4% paraformaldehyde at 4°C for more than 24 h. Tissues were processed, embedded in paraffin, sectioned at 4 mm, and stained with hematoxylin and eosin.

### Quantitative PCR analysis.

Fecal DNAs were extracted from fecal pellets (60 to 120 mg per sample) by using a TIANamp Stool DNA kit (Tiangen Biotech, catalog no. DP328). The relative abundance of *cutC*-positive bacteria and *Desulfovibrio* in the cecal content was estimated by qPCR analysis of the relative DNA copy numbers of the *cutC*- or *Desulfovibrio*-specific gene fragment in the extracted genomic DNAs. The primer sequences were as follows: *cutC* (F, 5′-TTYGCIGGITAYCARCCNTT-3′; R, 5′-TGNGGRTCIACYCAICCCAT-3′), bacterial 16S rDNA (F, 5′-CGGTGAATACGTTCCCGG-3′; R, 5′-TACGGCTACCTTGTTACGACTT-3′), and *Desulfovibrio* (F, 5′-TGGCAGATMATGATYMACGG-3′; R, 5′-GGGCCGTAACCGTCCTTGAA-3′).

Total RNAs from tissue were extracted by using TRIzol reagent (Ambion, catalog no. 15596026). cDNAs were synthesized using a high-capacity cDNA reverse transcription kit (TaKaRa, catalog no. RR047A). The qPCR was performed using the AceQ qPCR SYBR green Master Mix (Vazyme, catalog no. Q111) kit. The relative mRNA expression levels were determined by using the 2^–ΔΔ^*^CT^* method with *β-Actin* as the internal reference control, respectively. The primer sequences were as follows: *β-Actin* (F, 5′-TGAGAGGGAAATCGTGCGTGAC-3′; R, 5′-GCTCGTTGCCAATAGTGATGACC-3′), *G6pc* (F, 5′-CATCAATCTCCTCTGGGTGGC-3′; R, 5′-TGTTGCTGTAGTAGTCGGTGTCC-3′), *Pck1* (F, 5′-AAGCAAGACAGTCATCATCACCCAA-3′; R, 5′-GGCGAGTCTGTCAGTTCAATACCAA-3′), and *Foxo1* (F, 5′-ATGGTGAAGAGCGTGCCCTAC-3′; R, 5′-CTTTCCAGTTCCTTCATTCTGCAC-3′).

### Statistical analysis.

Data were analyzed with Prism (GraphPad Software) and are presented as means ± the standard errors of the mean (SEM). Statistical significance was determined by using an unpaired two-tailed Student *t* test for single variables and one-way analysis of variance (ANOVA) with Tukey’s *post hoc* tests or two-way ANOVA, followed by Bonferroni *post hoc* tests, for multiple variables. *P* values of <0.05 were considered statistically significant.

### Data availability.

The targeted UHPLC-MS/MS data will be provided upon request.
