# Supplementary material for: A Murine Commensal Protozoan Influences Host Glucose Homeostasis by Facilitating Free Choline Generation
Source: Appl Environ Microbiol. 2022 Mar 22;88(6):e02413-21. doi: 10.1128/aem.02413-21 (PMC8939315; doi:10.1128/aem.02413-21)
Supplement: Supplemental file 1 — Fig. S1 to S7. Download aem.02413-21-s0001.pdf, PDF file, 0.9 MB [file aem.02413-21-s0001.pdf]

Supplemental Material

A murine commensal protozoan influences host glucose homeostasis by facilitating free choline generation

Yanbo Kou[a1], Liyuan Meng[a1], Shenghan Zhang[a], Xingping Zheng[a], Mengnan Liu[a], Shihong Xu[a], Qiyue Jing[a], Hanying Wang[a], Jinzhi Han[a], Zhuanzhuan Liu[a], Yanxia Wei[a], and Yugang Wang[a#]

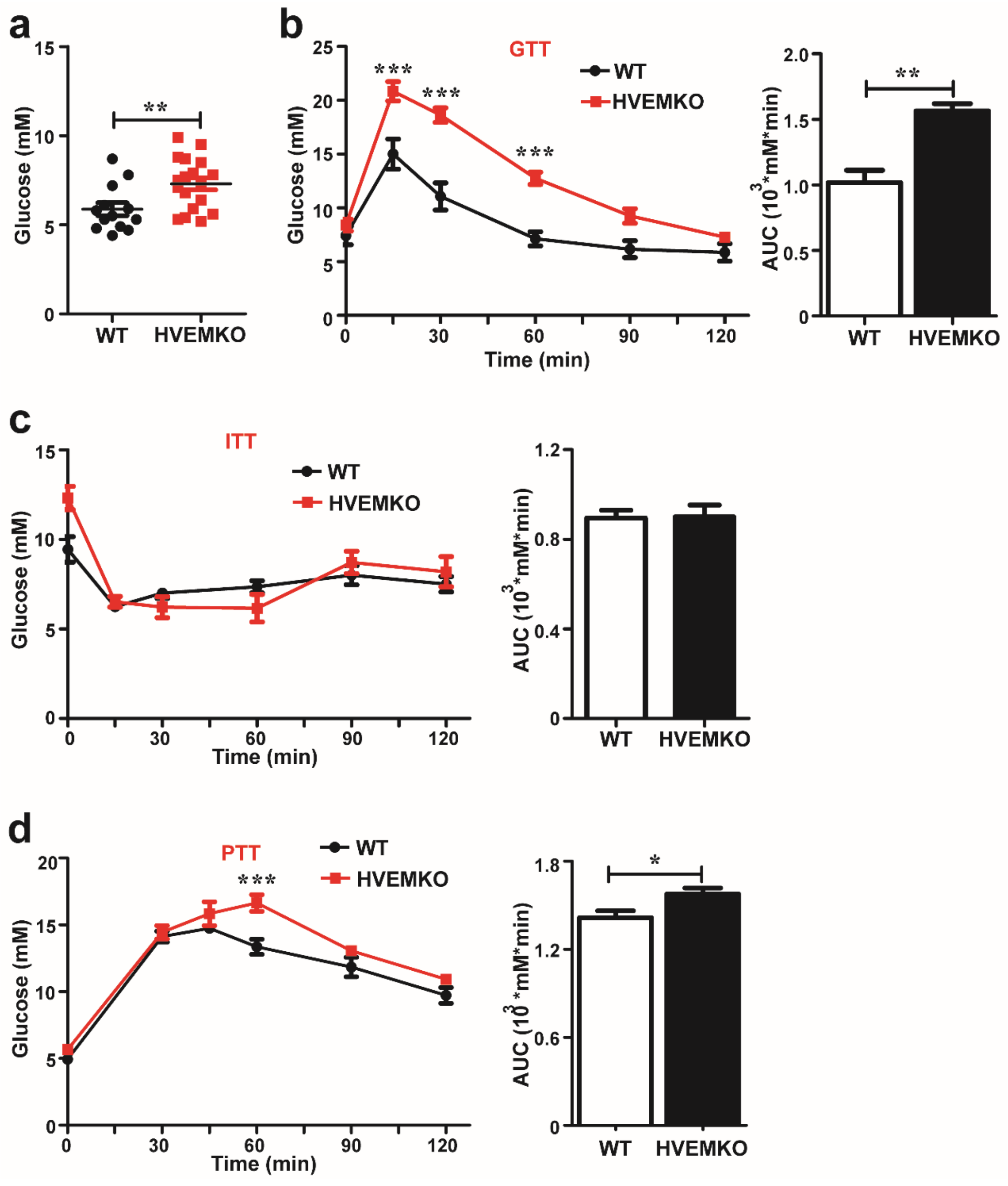

Supplementary Figure S1. The glucose metabolism in HVEM KO mice bred in-house is abnormal. (a) 8-week-old male wild-type (WT) obtained from VRL and HVEM KO mice bred in-house were fasted overnight. The plasma fasting glucose levels were then measured. N=13-18 mice/group. **(b)** Glucose tolerance testing (GTT) was performed. AUC, the area under the curve. N = 5 mice/group. **(c)** Insulin tolerance testing (ITT) was performed. N = 5 mice/group. **(d)** Pyruvate tolerance testing (PTT) was performed. N=3-5 mice/group. Data were represented as the mean ± SEM. * $p < 0.05$; ** $p < 0.01$; *** $p < 0.001$.

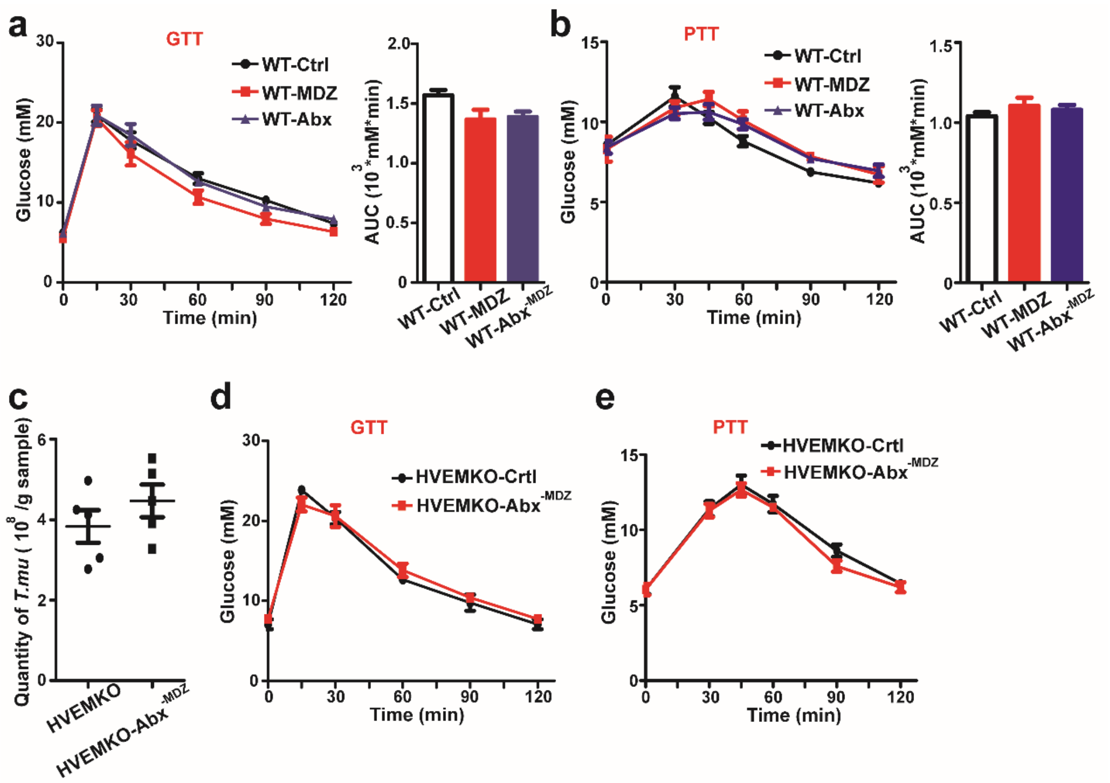

Supplementary Figure S2. The disturbed glucose homeostasis observed in HVEM KO mice was related to *T.mu* colonization. (a, b) *T.mu-free* WT mice from VRL were fed with vehicle control, metronidazole (MDZ), or a cocktail of broad-spectrum antibiotics lacking metronidazole (Abx^{-MDZ}, vancomycin, ampicillin, and neomycin) in drinking water for a week. Glucose tolerance testing (GTT) (a) and pyruvate tolerance testing (PTT) (b) were then performed. N=5-10 mice/group. **(c-e)** HVEM KO mice were fed with vehicle control or Abx^{-MDZ} in drinking water for a week. The number of *T.mu* presented in the cecal content was calculated (c), GTT (d) and PTT (e) were performed. N=4-5 mice/group. Data were represented as the mean ± SEM. * $p < 0.05$. AUC, the area under the curve.

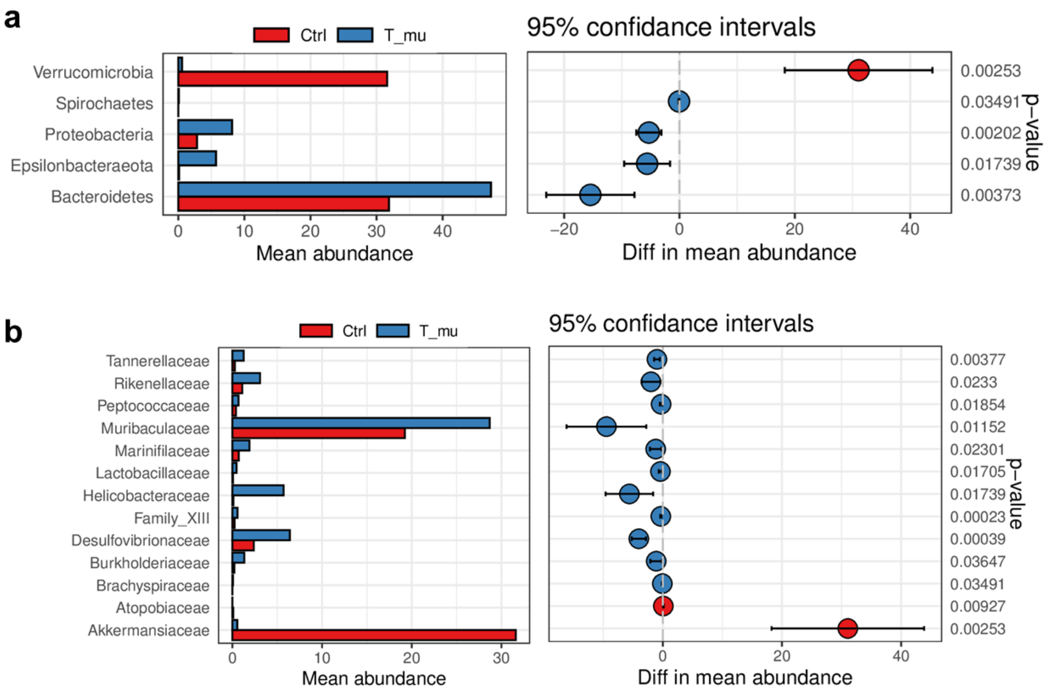

Supplementary Figure S3. *T.mu* colonization changed the gut microbiota landscape. (a, b) *T.mu-free* WT mice from VRL were administered with vehicle PBS control (ctrl) or *T.mu* (1×10^6/mouse) via oral gavage. One week later, cecal material was collected, bacterial genomic DNAs were extracted, and bacterial 16S rRNA gene sequences were then determined (N=5). The differentially enriched bacterial phyla (a) or families (b) in the indicated mice were shown.

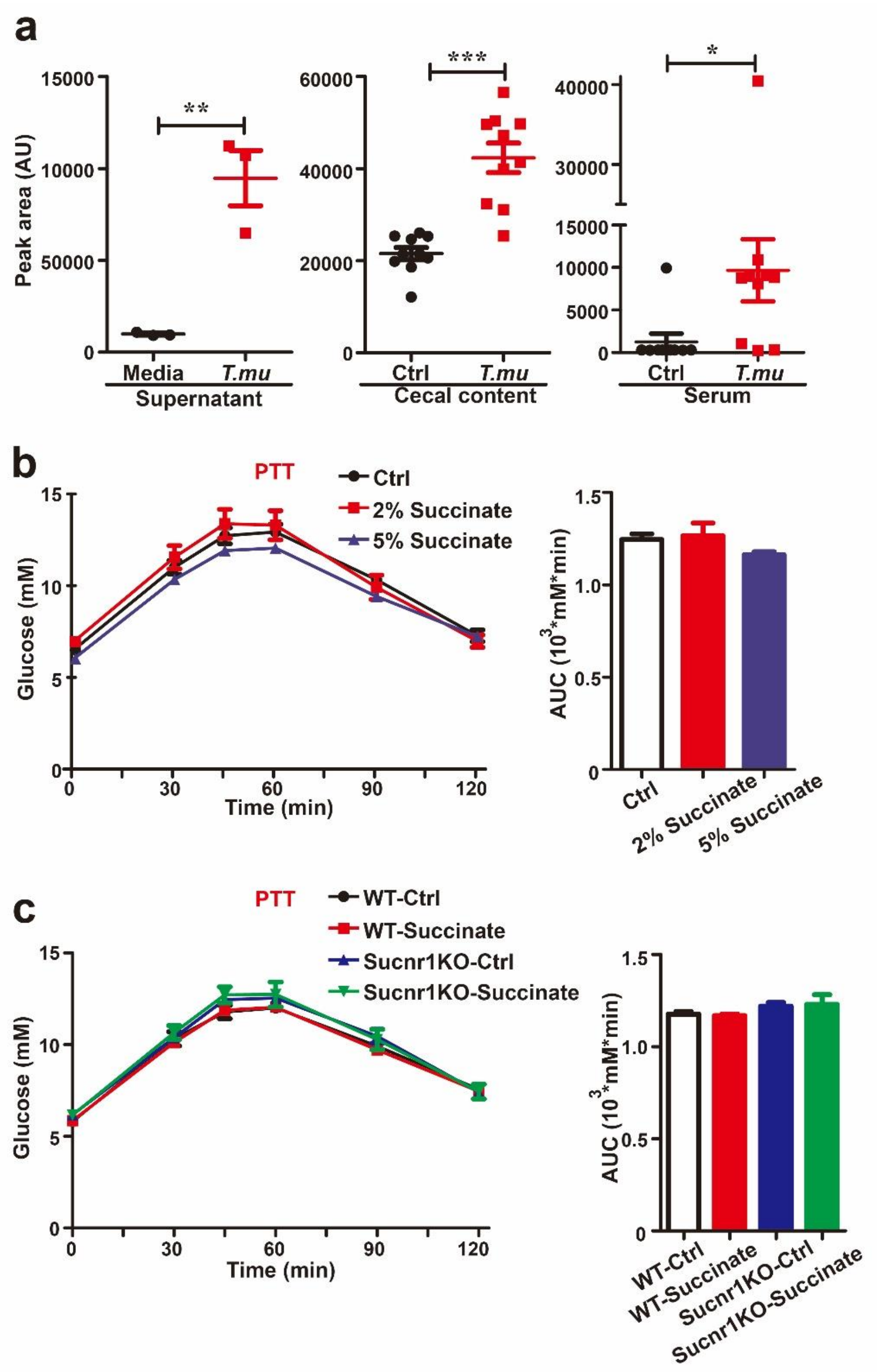

Supplementary Figure S4. Succinate does not enhance gluconeogenesis. (a) Succinate levels in *T.mu*-cultured supernatant (n=3/group), the cecal contents (n=10/group) and the serum (n=10/group) of *T.mu*-free control and *T.mu*-colonized WT mice. **(b, c)** WT (b) or succinate receptor Sucnr1 KO mice (c) were fed with/without disodium succinate in drinking water for 1 week, pyruvate tolerance testing (PTT) was then performed. N=5-10. Data were represented as the mean ± SEM. * $p < 0.05$; ** $p < 0.01$; *** $p < 0.001$. AUC, the area under the curve.

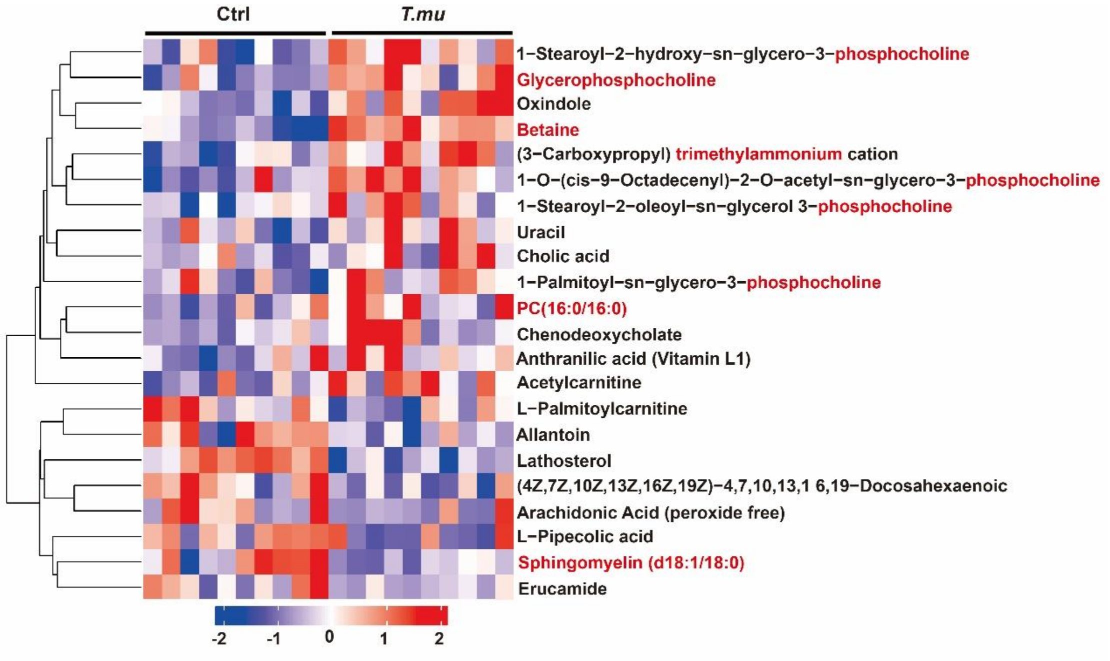

Supplementary Figure S5. *T.mu* colonization increases the serum levels of choline derivatives. Heatmap shows the relative levels of the differentially expressed metabolites in the serum of *T.mu*-free control (ctrl) versus *T.mu*-colonized WT mice. N=10 mice/group. Metabolites highlighted in red color are choline derivatives.

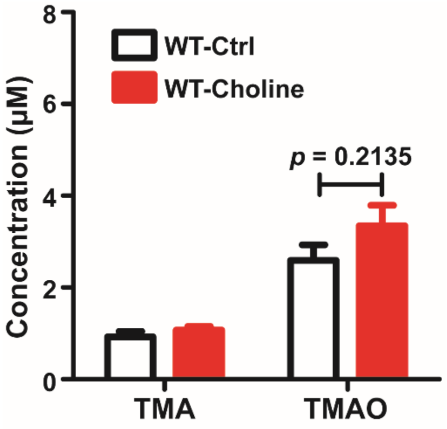

Supplementary Figure S6. Choline supplementation in WT mice did not increase serum levels of TMA and TMAO. 8-week-old male *T.mu*-free WT mice were treated with vehicle (ctrl) or 1 g/liter free choline in drinking water for 1 week. The serum levels of TMA and TMAO were then determined. N=10 mice/group.

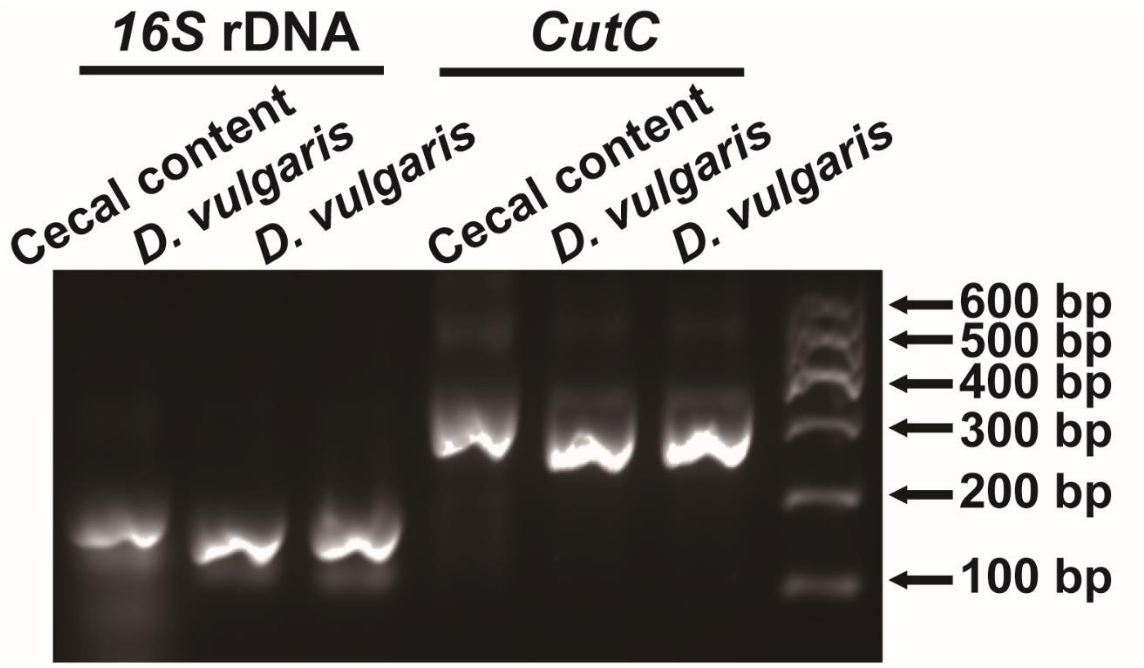

Supplementary Figure S7. *D. vulgaris* encodes the *cutC* gene. Bacterial genomic DNAs were extracted from *in vitro* cultured *D. vulgaris* or cecal content of WT mice and were then used as the template to amplify the *cutC* or 16S rDNA gene fragment by PCR.
